# Developing a Model for the Establishment of the Hospice Care Delivery System for Iranian Adult Patients With Cancer

**DOI:** 10.3389/fpsyg.2022.807621

**Published:** 2022-03-28

**Authors:** Samira Beiranvand, Maryam Rassouli, Maryam Hazrati, Shahram Molavynejad, Suzanne Hojjat, Kourosh Zarea

**Affiliations:** ^1^Nursing Care Research Center in Chronic Diseases, Ahvaz Jundishapur University of Medical Sciences, Ahvaz, Iran; ^2^Cancer Research Center, Shahid Beheshti University of Medical Sciences, Tehran, Iran; ^3^Community Based Psychiatric Care Research Center, Shiraz University of Medical Sciences, Shiraz, Iran; ^4^Department of Home-Based Palliative Care, ALA Cancer Prevention and Control Center (MACSA), Tehran, Iran; ^5^French Institute of International Research and High Education, Paris, France

**Keywords:** cancer, hospice, palliative care, health system, Iran

## Abstract

**Introduction:**

Making appropriate plans for the provision of hospice care is considered a perceived need in the Iranian health system. The current study aimed to develop a model for establishing hospice care delivery system for the adult patients with cancer.

**Materials and Methods:**

This study is part (data of the third and fourth phases) of a larger study that has been done in four phases. This Health System Policy Research (HSPR) utilized a mixed qualitative-quantitative approach. At the first phase, a qualitative study was conducted which explained the care needs and the requirements for establishing this system from the stakeholders’ perspective (directed content analysis). The second phase aimed to examine the current situation of hospice care delivery in Iran and identify and determine the similarities and differences among them in the selected countries (comparative study). At the third phase, the main areas of the model and the related indicators were extracted and prioritized by consulting with experts (Delphi survey). Then the model was formulated. At the fourth phase, the proposed model was validated and finalized in terms of importance, scientific acceptability, and feasibility.

**Results:**

Based on the developed model the first and the most important step in establishing the hospice care delivery system is laying the groundwork in the health system which requires focusing on policymaking. It is necessary to establish hospice centers and implement public awareness raising programs, train, and supply expert manpower, strengthen family physician and referral process, formulate clinical guidelines, encourage the private sector and NGOs(Non-Governmental Organizations (NGOs).) to invest, develop end-of-life and hospice care service packages, and create quality care indicators. The proposed model had moderate feasibility.

**Conclusion:**

This model was developed based on the current Iranian healthcare structure and the needs of terminally ill cancer patients. It can be used as a model tailored to the current state of the health system and community in Iran. It is suggested to use this model as a pilot at the regional level.

## Introduction

Cancer is a chronic disease with a rising prevalence. According to the WHO reports in 2018, the incidence rate of cancer has reached 18.1 million cases globally. Furthermore, according to this report, the latest cancer-related mortality rate is almost 9.6 million cases worldwide (World Health Organization, 2018b). In Iran, the prevalence of cancer is increasing progressively and is known as the second leading cause of death. In 2016, 82% of deaths in Iran were related to chronic diseases, with cancer accounting for 16% of them (Bray et al., 2018).

Advancements in technology and pharmacotherapy has often led to an increased number of patients with chronic diseases including cancer, and the long-term survival of these patients, which will increase the need for end-of-life and death care (Kumar et al., 2017). More than half of cancer patients are hospitalized at the time of death and undergo highly invasive procedures that do not necessarily lead to better outcomes for the patient and family and may not even be consistent with their preferences, while these patients only need end-of-life care (Wright et al., 2016).

The lack of a formulated system for addressing the primary needs of these patients will result in unnecessary and frequent visits to the ERs of specialty and subspecialty centers, hospitalization in ICUs^1^, burdening patients with expensive examinations and tests and, in some cases, taking unnecessary treatment measures (Amiresmaeili et al., 2015; Angus and Truog, 2016).

In addition, the lack of formal and structured training, insufficient knowledge, and experience in providing specific end-of-life care, the sense of providing futile care, mental pressures, and ethical problems and issues is among the challenges nurses and other members of the care team face while providing care for these patients in hospitals’ Ers^2^ and ICUs (Valiee et al., 2012; Borhani et al., 2014; Rassouli and Sajjadi, 2016). Family caregivers encounter many problems and are concerned about safe care, too (Nemati et al., 2018). Therefore, the hospital is not considered a proper place to provide end-of-life services.

In many communities, this type of care is provided by hospice centers and specialty end-of-life care teams (Clark et al., 2014). Hospice centers are defined as settings for providing out of hospital palliative care services or, in other words, at the community level, which are considered to be essential in some countries for addressing the needs of the people with reduced capacity who are at the end-of-life stage (Hui, 2014). Care services at hospice centers focus on the provision of the best possible quality of life for end-of-life patients and their families by preventing and relieving their physical, emotional, social, and spiritual suffering (National Hospice Palliative Care Organization, 2020). Despite this necessity, the available data shows large deficiencies in the provision of and access to such services in many low- and middle-income countries (World Health Organization, 2018a). The results of the studies conducted in Iran, as in other countries, show that critically ill and end-of-life patients are generally hospitalized in ICUs and extremely large sums are spent for them (Ziloochi et al., 2012; Amiresmaeili et al., 2015). There are no hospice centers in Iran, and the conducted research have focused on setting up and establishing centers specifically for providing end-of-life care and hospice services (Valiee et al., 2012; Borhani et al., 2014; Azami-Aghdash et al., 2015; Ansari et al., 2019).

According to National Action Plan for Prevention and Control of Non-Communicable Diseases, the plans developed for the next two decades should focus on the specific needs of those with the most common chronic diseases, especially cancer. As the demand for end-of-life care increases, patients and their families seek optimal care (Borhani et al., 2014). At the same time, considering the increased rate of chronic diseases, especially cancer, and the aging of the population, and based on the successful experiences of the developed countries in this aera, the designing and development of hospice services in Iran seems necessary. However, the provision of these services depends on various factors, including the cultural and social status of community (Azami-Aghdash et al., 2015). In this regard, a study, titled as *Developing a Hospice Care Delivery System for Iranian adult patients with cancer*, was conducted using a mixed qualitative-quantitative research approach. By adopting a mixed approach, the research team tried to design and propose a local model of hospice care provision system for the adult patients with cancer across the country. So, the present study Contains results of the third and fourth phases of the main study that was done with the following aims.

1. extracting and prioritizing the main areas of the model and the related indicators by consulting with experts (phase 3).
2. formulating the model and validating it in terms of importance, scientific acceptability, and feasibility.

## Conceptual Framework of the Research

The present study was designed and conducted based on the Hospice Palliative Care System Design Framework (2010). This framework is specifically developed by Ontario’s Regional End of Life Care Networks (EOLCN) and Hospice Palliative Care Networks (HPCN) for designing regional hospice care systems and evaluates 6 domains: (1) care settings and service, (2) programs within care settings and services, (3) integration/linkages, (4) human resources, (5) accountability, and (6) policies, guidelines, and funding (Seniors Health Research Transfer Network, 2012). In each domain, the requirements, and the standards for designing a hospice palliative care delivery system are discussed in detail. This framework describes and classifies the essential domains and elements of an integrated hospice palliative care system and shows the essential and favorable parts of each domain separately.

## Methodology

**FIGURE 1:**
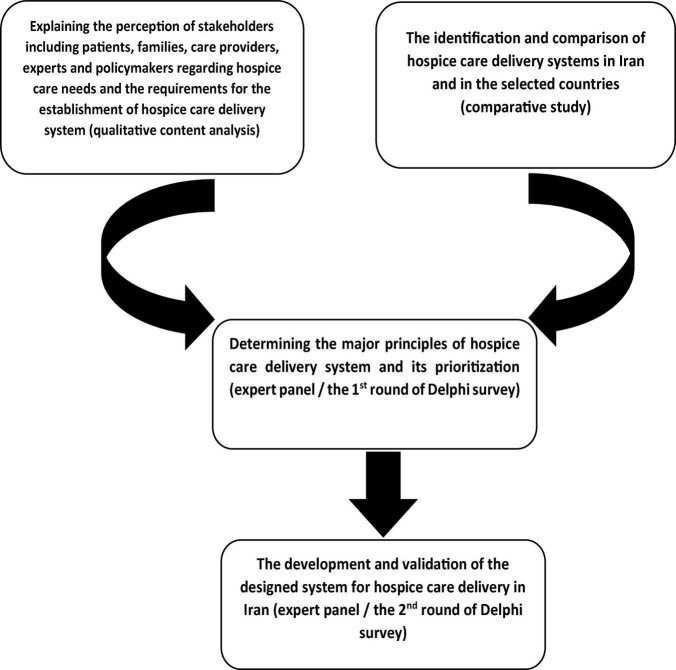
Phases of the research.

This is a Health System Policy Research (HSPR) (Hojjat-Assari et al., 2021) conducted using a sequential mixed method in 4 phases from February 2018 to July 2020. Figure 1 displays the phases of the study.

### The First Phase

In this phase (from February to November 2019), a qualitative study was done using the directed content analysis to explain patients, families, caregivers, specialists, experts, and policymakers’ perceptions of the care needs of cancer patients in the end-of-life stages, and the requirements for establishing a hospice care delivery system. The participants were selected through purposive sampling which continued until data saturation was achieved. Some of the samples were selected using snowball sampling. Data was collected using semi-structured interviews with open-ended questions. The results of this phase are also accepted for publication in the International Journal of Palliative Nursing. But not yet online.

### The Second Phase

This phase (from December 2019 to April 2020) aimed to review and analyze the background and the *status quo* of hospice care delivery in Iran and tried to identify and compare the existing models of hospice care delivery for the adult patients with cancer across the world in the form of a comparative study. The hospice care systems of the United Kingdom, Canada, Australia, Japan, India, Jordan, and Iran were chosen for further investigation as target samples based on the reports by Economist Intelligence Unit (EIU). The search was conducted using keywords extracted from Medical Sub Headin (MeSH) in databases both national (SID, Magiran and Iran Medex) and international (Scopus, PubMed, Web of sciences, ProQuest, CINAHL, MedlinePlus, EMBASE, Cochrane Library, and Google Scholar), and prominent specialty journals in the field of hospice and palliative care, scientific and administrative documents, WHO website and reports, governmental websites and other official sites, important national and regional websites related to organizations active in the field of cancer, hospice and palliative care and the data provided by the websites of at least three hospice centers in each country were all reviewed in detail (Zarea et al., 2020).

### The Third Phase

The purpose of this phase (from May to August 2020) is to combine the findings of the previous phases of the study, to extract the main components related to each domain for designing and developing a regional model of hospice care delivery system for adults with cancer in Iran, and to prioritize them. Therefore, after combining the results of the first and the second phases of the study, and identifying the main domains for model development, a questionnaire consisting of 127 items (components based on the main domains and subdomains of the model) was designed in seven parts. In fact, each part of the questionnaire was designed for each extracted domain from the results of qualitative and quantitative studies in the first and second phases. Each section contained several questions which were also based on the most important components of the model related to each domain. Given that seven domains were extracted from the first and second stages of the study, therefore, the questionnaire consisted of seven sections. Likert scale ranging from *very high* (4), *to low* (1) was used. The questionnaire provided to a group of experts for prioritization based on Delphi survey. The Delphi panelists were selected in this phase of the research, through purposive sampling and the snowball sampling, from among the health system policymakers and the physicians with competences in the field of cancer and palliative care, university faculty members and nursing professors with competences of teaching theoretical and practical courses related to cancer, and palliative care and other health care workers such as nurses, psychologists, occupational therapists, and nutritionists with more than 1 year experience of working with end-stage cancer patients. It should be noted that some of the specialists and experts were selected from among charity donors and the officials of NGOs, charitable organizations, and the Health Insurance Organization. In this study, according to the vast scope of the subject and the available resources as well as similar previous studies (Habibi et al., 2014), after the consent of 21 experts was obtained for participation in the research, the questionnaire *A Survey on the Importance of the Domains of Designing Hospice Care Delivery System for adult patients with cancer* were provided for them through in-person visits or *via* e-mail. The participants were asked to prioritize the importance of each item of the questionnaire based on their own perspective and competences, ranging from *very high* (4), *high* (3), *medium* (2), and *low* (1). Furthermore, there was an open-ended question at the end of the questionnaire, where they could express their other opinions in regard with the different parts of the questionnaire. The Decision Delphi was used in this study with a quantitative approach as well as the 10 steps proposed by Fawle (Rauch, 1979) to implement the Delphi survey. To this end, the data was analyzed in this phase using descriptive statistics through SPSS software, and the mean value and the standard deviation were calculated for the score of each item. Then, based on the approaches of similar studies (Ansari, 2018), items with a mean score of 3 and above were extracted. Also, to determine the degree of importance of each dimension, the standardized score of each dimension was calculated using the minimum and maximum scores of that dimension. By the end of this phase, a draft of the hospice care delivery system model for cancer patients was developed.

### The Fourth Phase

Finally, the Phase 4 (from September to December 2020)of the study was conducted again with the aim of assessing the credibility of the developed model in term of feasibility using the Delphi survey. It should be noted that this phase of the research is the 2nd round of Delphi survey. As a result, due to the participation of the same subjects in both stages, some previously explained characteristics of the panelists are not mentioned in this phase. For this purpose, after removing some items with a mean score below 3 in the previous phase, a 109-item questionnaire containing the 7 domains were developed and offered to the experts. The participants were asked to separately rate the feasibility of each of the components as *low* (1), *medium* (2) and *high* (3). The model of hospice care delivery system for cancer patients was then finalized, after receiving the comments and the suggestions and applying them. In this phase, SPSS and descriptive statistics were used to investigate how to answer the questionnaire items.

### Ethical Consideration

This paper was a part of a PhD dissertation in nursing at the Chronic Disease Care Research Center affiliated to the Ahvaz Jundishapur University of Medical Sciences (grant No. NCRCCD9709). The ethics approval for the research was obtained from the Ahvaz Jundishapur University of Medical Sciences under the code of IR.AJUMS.REC.1397. 306. The authors have no conflicts of interest.

## Findings

### The Results of Phase 1

At this stage, according to the research objectives and the inclusion criteria, 21 key experts including 3 cancer patients, 6 family caregivers, 8 caregivers, and 4 policymakers were interviewed. Finally, 7 main domains, 18 sub-domains, and 55 sub-subdomains were extracted from the analysis of the qualitative data. The results along with some more details are accepted to the International Journal of Palliative Nursing as a paper, and it will be published soon.

### The Results of Phase 2

Based on the results of the comparative study, it was discovered that Iran does not have any integrated and formal structure or program for the provision of hospice services, and even end-of-life services. Of course, in recent years, few centers have provided sporadic palliative care services to patients with life-threatening conditions, especially those with cancer, in inpatient wards and homes, mainly in large cities, which contained end-of-life care. These services are provided without referring to clinical guidelines and solely based on caregivers’ experiences and knowledge and are far from global standards. In addition, based on the comparison of hospice care system in the selected countries (United Kingdom, Canada, Australia, Japan, India, and Jordan), it can be said that successful and leading countries, despite the establishment of hospice centers, try to expand this type of service into all areas of the health system. In order to establish and develop hospice services in the above countries, important measures have been taken including having a strong and effective policymaking framework and comprehensive national programs, the integration of services into the palliative care system and, at a higher level, in the health system, government commitment to funding services, training specialized manpower, financial plans to help families and caregivers, adopting essential laws and regulations, the development of clinical protocols and guidelines, providing access to essential drugs and narcotics, increased public awareness and expanding research. The results of the first phase are already published in detail (Rassouli and Sajjadi, 2014, 2016; Mojen et al., 2017; Zarea et al., 2020).

### The Results of Phase 3

The preliminary draft of the model for establishing hospice care delivery system was developed as a questionnaire. Out of 21 questionnaires distributed among the participants in Phase 3 (1st round of Delphi), 18 were fully completed and were then analyzed. The results showed that 10 men and 8 women with the mean age of 44.33 ± 15.67 years and 12.11 ± 9.89 years of work experience participated in the study. Table 1 shows the personal characteristics of the participants in this phase.

**TABLE 1:** The demographic characteristics of participants in Phase 3.

No	Gender	Age (years)	Working experience (years)	Specialty	Education
1	Male	45	12	Faculty member	PhD in nursing
2	Male	39	10	Instructor	BSN
3	Female	45	16	Faculty member	PhD in nursing
4	Male	30	7	Oncology nurse	MSc in nursing
5	Male	38	5	Palliative nurse	BSc in nursing
6	Female	35	3	Oncology psychologist	PhD in clinical psychology
7	Male	60	24	Faculty member	PhD in health policy
8	Male	50	15	Surgical oncologist	Specialist
9	Male	55	20	Oncologist	Specialist
10	Female	42	13	Faculty member	PhD in nursing
11	Female	50	18	General practitioner	MD
12	Male	46	10	General practitioner	MD
13	Female	48	13	Public relations postgraduate	Social worker
14	Female	28	6	Spiritual therapist	PhD in theology
15	Male	55	23	Faculty member	PhD in health economics
16	Male	41	10	Epidemiologist	MD
17	Female	48	5	General practitioner	MD
18	Female	38	8	Lymphatic therapist	MD

After data analysis, the subjects obtained scores from 2.15 ± 0.87 to 4. Of these, the score of 18 questions were between 2.15 ± 0.87 and 2.91 ± 0.9, which were then excluded. Of these 18 questions, 4 questions were in the need to hospice center in home, hospital, outpatient clinics, and long -term care centers, 1 question was in the level of referral, 6 questions were about the specialist interdisciplinary team, 3 question about the skill and academic training/expected competencies, 1 question about the transparent decision making and 3 questions about the research development. Since the questionnaire contained a lot of details and was almost complete, most panelist did not have a specific comment for this part. In Table 2 shows how the findings of the first phase (the comparative study) and the second phase (the qualitative study) are combined. Some components are only obtained from the findings of the comparative study; some, from the results of the qualitative study; and most of them, from both. The components were placed in a questionnaire. As well as the mean and standard deviation of the scores assigned by the experts has been reported. After removing the items with the mean scores below 3, 102 indicators remained. According to experts, “*Organizing Accountability*,” “*Integration into The Health System*,” “laying the groundwork *in the health system*” with a degree of importance of 93%, 88%, 85% respectively, are the most important domains in the design and deployment of the model. “*comprehensive care plan*” (83%), “*capacity building in the community*” (79.5%), “*the need to provide a variety of settings and services*” (79.2%), and “*specialized manpower*” (76%) were respectively in the next degree of importance.

**TABLE 2:** The main domains and components extracted from the findings of Phase 1 (comparative study) and Phase 2 (qualitative study) and the mean, minimum and maximum score of components in Phase 3 (minimum mean score = 1, maximum mean score = 4).

Domains	Subdomains	Components	Based on		Mean ± SD
			Comparative study	Qualitative study	
**The need to provide a variety of settings and services**	The need for hospice centers	The establishment of hospice centers	*	*	3.9 ± 0.85
		Services provision by hospice centers at home	*		2.91 ± 0.9
		Services provision by hospice centers at hospital	*		2.15 ± 0.87
		Services provision by hospice centers at outpatient clinics	*		2.91 ± 0.9
		Services provision by hospice centers at long-term care centers	*		2.15 ± 0.87
	Comprehensive patient/family centered services	Managing physical symptoms of the end stage patient	*	*	4
		Managing mental symptoms of the end stage patient	*	*	4
		Providing spiritual care to the end-stage patient and family caregivers	*	*	3.3 ± 0.75
		Addressing the mental-spiritual needs of the family until the end-stage patient passes away	*	*	3.8 ± 0.75
		Addressing the social needs of the patient and family until the end-stage patient passes away	*	*	3.3 ± 0.75
		Providing bereavement care to the family after patient’s death	*	*	3.5 ± 0.7
		The training of end-stage patient and family caregivers	*	*	3.9 ± 0.87
		Offering the necessary information to the end-stage patient and family caregivers	*	*	4
		Considering the care ability of family caregivers while entrusting the end-stage patient to them		*	3.6 ± 0.85
**Comprehensive care program**	Participatory decision-making	End-stage patient’s involvement in decision making regarding the place of death and care receiving	*		3.2 ± 0.64
		Family’s involvement in decision making regarding the place of death and end-stage patient’s care receiving	*		3.3 ± 0.81
		Respecting end-stage patient’s right to know his/her own state	*	*	3.8 ± 0.78
		Respecting family’s right to know the state of the end-stage patient	*	*	4
		Supporting end-stage patient in making his/her decision regarding self	*	*	4
		Supporting family in making decision regarding the end-stage patient	*	*	4
		Attention to physician’s decision at patient’s end-of-life stage	*	*	3.3 ± 0.75
		Attention to the end-stage patient’s religious-cultural issues		*	3.7 ± 0.81
		Attention to the religious-cultural issues of the family caregivers of end-stage patient		*	3.7 ± 0.81
	Consensus on the definition of the procedure	The need to define hospice care according to the WHO’s definition		*	3.3 ± 0.75
		The need to set a standard referral time for receiving hospice care	*	*	3.9 ± 0.8
		Setting the duration of hospice service delivery	*		3.8 ± 0.78
		Setting precise admission criteria for receiving hospice services	*	*	3.8 ± 0.78
		Comprehensive assessment of end-stage patient’s needs	*	*	3.7 ± 0.78
		Comprehensive assessment of the needs of the family with end-stage patient	*	*	3.6 ± 0.80
		Developing a care plan based on the end-stage patients and family	*	*	3.5 ± 0.78
		Precise explanation of hospice care delivery procedure (assessment, follow up, visits, etc.)	*	*	4
		24/7 phone consultation to the family of end stage patient regarding care	*		3.5 ± 0.76
		Establishing communication between family and hospice care team during patient’s life and after death	*	*	3.6 ± 0.85
		Formulating guidelines for referral and transfer between different settings (hospital, home, etc.) if necessary	*	*	4
		Formulating visitation guidelines in the care plan at various settings	*	*	3.5 ± 0.78
		Formulating standard diagnostic and treatment guidelines	*	*	3.5 ± 0.78
		Formulating standard guideline for pain management in end-stage patient	*	*	4
		Formulating standard guideline for symptom management in end-stage patient	*	*	4
		Assessing the effectiveness of the training of family caregivers	*		3.30 ± 0.80
		Care quality assessment	*		3.5 ± 0.78
**Integration into the health system**	Integration of services	The integration of hospice care services at the primary level (outpatient clinics, urban health centers and comprehensive health centers)	*	*	3.5 ± 0.75
		The integration of hospice care services at the secondary level (general and specialty hospitals)	*	*	3.8 ± 0.81
		The integration of hospice care services at the tertiary level (home, long-term care centers)	*	*	3.4 ± 0.75
	Level of referral	Patient’s referral by a family physician for receiving hospice services	*	*	3.5 ± 0.75
		Patient’s referral by a nurse for receiving hospice services	*	*	3.8 ± 0.81
		Patient’s referral by a general practitioner for receiving hospice services	*		3.7 ± 0.78
		Patient’s referral by an oncologist for receiving hospice services	*	*	4
		Patient’s referral by other member of the care team for receiving hospice services	*		2.9 ± 0.9
	Connection with other care settings	Establishing connection between hospice centers and other care settings (outpatient clinics and urban health centers, hospitals, and home)	*	*	3.8 ± 0.81
		Service coordination among different levels	*	*	3.9 ± 0.8
		Providing hospice centers with access to care and treatment documents	*	*	4
		Creating a national hospice care network to record the data of patients receiving care	*		3.5 ± 0.75
		The connection of hospice care national network to the electronic health record system (SEPAS)		*	3.9 ± 0.8
**Specialist manpower**	Specialist interdisciplinary team	Providing interdisciplinary and team care	*	*	4
		Training specialist hospice care delivery teams	*	*	4
		The presence of a pain specialist in the hospice care delivery team	*		2.9 ± 0.68
		The presence of a specialist nurse in the hospice care delivery team	*	*	4
		The presence of a nurse as a coordinator of team care	*		4
		The presence of a psychologist in the hospice care delivery team	*	*	4
		The presence of a social worker in the hospice care delivery team	*	*	4
		The presence of a nutritionist in the hospice care delivery team	*	*	2.9 ± 0.63
		The presence of a spiritual caregiver/cleric in the hospice care delivery team	*	*	4
		The presence of a physiotherapist in the hospice care delivery team	*	*	2.9 ± 0.63
		The presence of an occupational therapist in the hospice care delivery team	*		2.3 ± 0.79
		The presence of a general practitioner in the hospice care delivery team	*	*	3.9 ± 0.81
		The presence of an oncologist in the hospice care delivery team	*	*	2.9 ± 0.60
		The presence of volunteer workforce in the hospice care delivery team	*		2.9 ± 0.60
	skill and academic training/Expected competencies	Including the concept of hospice care in the curriculums of undergraduate nursing program and general practice	*	*	3.9 ± 0.81
		Including the concept of hospice care in the curriculums of the undergraduate programs in other related disciplines	*	*	3.9 ± 0.78
		The need to develop hospice care specialized discipline	*	*	2.9 ± 0.63
		The need to develop hospice care fellowship course	*	*	2.9 ± 0.63
		The need to develop a master program for hospice care	*	*	2.7 ± 0.68
		Holding hospice care short-term skill training courses	*	*	4
		Holding ongoing training courses for the staff	*	*	3.9 ± 0.81
		Holding periodic hospice care workshops	*	*	4
		Providing the necessary educational settings for training specialist manpower	*	*	4
		Providing standard educational content for training specialist manpower	*	*	4
		The involvement of related organizations in hospice care training	*	*	3.8 ± 0.78
		Defining credible specialized licenses and certificates for the trained manpower	*	*	3.9 ± 0.81
		Defining the competencies expected from the members of the hospice care team	*	*	3.8 ± 0.78
		Educational planning based on the competencies expected from the members of the hospice care team	*		3.8 ± 0.78
		Considering the topics related to hospice care in professional competence exams of nurses and physicians and other disciplines	*		3.2 ± 0.64
**Organizing the accountability system**	Transparent decision-making	Developing the hospice care delivery system for various types of adult cancer	*		2.9 ± 0.63
		Creating a joint taskforce for hospice care policymaking at the level of the Ministry of Health	*	*	3.9 ± 0.81
		Creating a joint taskforce for hospice care at the university level	*	*	3.9 ± 0.81
		Creating a joint taskforce for hospice care at the level of specialty hospitals	*	*	3.7 ± 0.68
		The participation of all sectors involved in the development of hospice care system	*	*	4
		The participation of governmental organizations in the development of hospice care system	*	*	4
		The participation of private organizations (charities, NGOs, etc.) in the development of hospice care system	*	*	3.9 ± 0.81
		The formulation of job descriptions, roles, responsibilities, and jurisdiction of each sector	*	*	3.8 ± 0.78
	Accountable management	The presence of a system for monitoring, assessment, and supervision	*	*	3.8 ± 0.78
		Developing indicators for monitoring, supervision, and accreditation	*	*	3.9 ± 0.81
		Launching the electronic document system and the access to database to make decisions in the field of policymaking	*	*	3.9 ± 0.81
**Laying the groundwork *in the health system***	Optimal policymaking	Providing documents related to the importance of hospice care for health policymakers	*	*	3.9 ± 0.81
		The obligation of government and the Ministry of Health to implement hospice care plan	*	*	3.4 ± 0.75
		Developing the national hospice care plan	*		3.8 ± 0.78
	Financial prerequisites	Government funding	*	*	4
		Funding by private organizations	*	*	3.9 ± 0.81
		Developing insurance service packages	*	*	4
		The involvement of charitable organizations in funding hospice services	*	*	3.9 ± 0.81
		Cost determination for secondary insurance services	*	*	3.7 ± 0.68
		Cost determination for governmental insurance services	*	*	4
		Cost determination for services separately for each discipline	*	*	3.8 ± 0.81
		Supporting private sector in service provision	*	*	3.9 ± 0.80
		Insurance supportive policies for the proper insurance coverage of medications	*	*	4
	Structural prerequisites	Providing infrastructure (resources, space, etc.)	*	*	3.9 ± 0.81
		Providing care equipment (ventilator, suction machine, oxygen, etc.)	*	*	4
		Developing palliative care network (service delivery at outpatient clinics, home-care centers, hospital, etc.)	*	*	3.8 ± 0.78
		Access to center data registry	*	*	3.4 ± 0.75
	Legal prerequisites	Access to a variety of necessary narcotic drugs	*	*	4
		Formulating guidelines for access to necessary narcotic drugs	*	*	4
		Formulating drug packages at hospice centers	*	*	3.9 ± 0.81
		The possibility for other disciplines such as nursing, pharmacy, etc. to prescribe drugs	*		3.4 ± 0.75
		Solving legal issues surrounding drug prescription (which drugs by whom?) at hospice centers	*	*	4
		Developing monitoring and supervisory indicators for supervision on and monitoring of pharmacotherapy at hospice centers	*		3.6 ± 0.73
		Formulating guidelines and instructions in accordance with the law, ethics, and scientific references in regard with continuing or stopping CPR	*	*	3.8 ± 0.81
		Determining the legal procedure related to hospice care	*	*	4
		Clarifying legal issues regarding hospice care	*	*	4
		Formulating supervisory rules for care at hospice centers	*	*	4
		Formulating supportive rule for care providers at hospice centers	*	*	4
**Capacity building at the community**	Raising public awareness	Holding training courses at the community level	*	*	4
		Providing the cultural and social infrastructure with the aim of increasing hospice care acceptability	*	*	4
	Research development	Launching interdisciplinary research centers related to hospice care	*		2.9 ± 0.63
		Publishing specific journals in the field of cancer hospice care	*		2.9 ± 0.63
		The participation of other organizations in conducting research on hospice care	*		2.7 ± 0.68
		Holding annual hospice care seminars and conferences	*		3.2 ± 0.63
		Determining the cost-effectiveness of hospice services	*	*	4

### The Model for Establishing Hospice Care Delivery System

**FIGURE 2:**
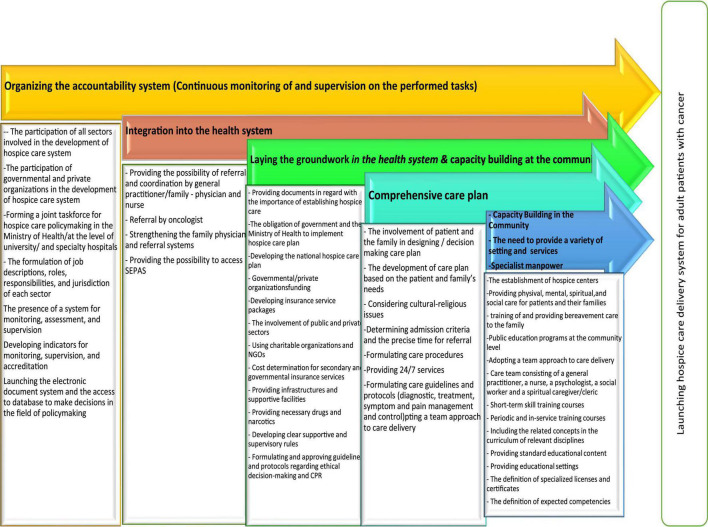
The model for the establishment of hospice care delivery system for adult patients with cancer.

Figure 2 show the proposed model for hospice care delivery system for adult patients with cancer and its dimensions that were adjusted to the upstream rules of Iranian health system. Dimensions are arranged according to the degree of importance in the opinion of experts. In addition, inside each box, there are components (activities) that are related to that dimension and are necessary for the establishment of a hospice care system.

### The Results of Phase 4

Out of the 18 questionnaires submitted in the 2nd round of Delphi, only 12 complete questionnaires were received. Table 3 shows the demographic characteristics of the participants.

**TABLE 3:** The demographic characteristics of participants in Phase 4.

No	Gender	Age (years)	Working experience (years)	Specialty	Education
1	Male	45	12	Faculty member	PhD in nursing
2	Male	39	10	Instructor	BSN
3	Female	45	16	Faculty member	PhD in nursing
4	Female	30	7	Oncology nurse	MSc in nursing
5	Female	38	5	Palliative nurse	MSc in nursing
6	Male	55	23	Faculty member	PhD in health policy
7	Male	50	15	Surgical oncologist	Specialist
8	Male	55	20	Oncologist	Specialist
9	Female	42	13	Faculty member	PhD in nursing
10	Female	50	18	General practitioner	MD
11	Male	46	10	General practitioner	MD
12	Male	55	23	Faculty member	PhD in health economics

The statistical results of the components in this phase of the research are shown in Table 4. Panelists did not have a specific comment for this part too. In general, the results show only the low feasibility of model in some components.

**TABLE 4:** The mean, minimum and maximum score of the Feasibility of components in Phase 4 (minimum mean score = 1, maximum mean score = 4).

Domains	Subdomains	Components	Feasibility (Mean ± SD)
**The need to provide a variety of settings and services**	The need for hospice centers	The establishment of hospice centers	2.91 ± 0.31
	Comprehensive patient/family centered services	Managing physical symptoms of the end stage patient	2.91 ± 0.31
		Managing mental symptoms of the end stage patient	2.91 ± 0.31
		Providing spiritual care to the end-stage patient and family caregivers	2.91 ± 0.31
		Addressing the mental-spiritual needs of the family until the end-stage patient passes away	2.85 ± 0.60
		Addressing the social needs of the patient and family until the end-stage patient passes away	2.42 ± 0.40
		Providing bereavement care to the family after patient’s death	2.85 ± 0.63
		The training of end-stage patient and family caregivers	2.85 ± 0.63
		Offering the necessary information to the end-stage patient and family caregivers	2.89 ± 0.70
		Considering the care ability of family caregivers while entrusting the end-stage patient to them	2.89 ± 0.70
**Comprehensive care program**	Participatory decision-making	End-stage patient’s involvement in decision making	2.73 ± 0.50
		Family’s involvement in decision making	2.85 ± 0.60
		Developing care based on patient’s needs	2.85 ± 0.80
		Developing care based on the family’s needs	2.89 ± 0.70
		Considering patient and family’s cultural issues	2.81 ± 0.78
	Consensus on the definition of the procedure	The definition of hospice care according to the WHO’s definition	2.55 ± 0.60
		Setting a standard referral time for receiving hospice care	2.89 ± 0.70
		Setting the duration of hospice service delivery	2.73 ± 0.50
		Setting precise admission criteria for receiving hospice services	3
		Comprehensive assessment of the patient and family’s needs	2.85 ± 0.60
		Developing a care plan based on the needs of end-stage patients and family	2.58 ± 0.80
		Precise explanation of hospice care delivery procedure (assessment, follow up, visits, etc.)	2.89 ± 0.70
		24/7 phone consultation and services	2.35 ± 0.40
		Formulating visitation guidelines in the care plan	3
		Formulating guidelines for admission, referral, and transfer between different settings (hospital, home, etc.)	3
		Formulating standard diagnostic and treatment guidelines	3
		Formulating standard guideline for pain management	3
		Formulating standard guideline for symptom management	3
		Establishing communication between family and hospice care team during patient’s life and after death	2.89 ± 0.70
		Identifying the stages of family bereavement and providing a care plan	2.73 ± 0.50
		Assessing the effectiveness of the training of family caregivers	2.58 ± 0.80
		Care quality assessment	2.42 ± 0.80
**Integration into the health system**	Integration of services	The integration of hospice care services at the primary level (outpatient clinics, urban health centers and comprehensive health centers)	2.34 ± 0.63
		The integration of hospice care services at the secondary level (general and specialty hospitals)	2.91 ± 0.31
		The integration of hospice care services at the tertiary level (home, long-term care centers)	2.91 ± 0.31
	Level of referral	Patient’s referral by a family physician/general practitioner for receiving hospice services	2.64 ± 0.84
		Patient’s referral by a nurse for receiving hospice services	2.56 ± 0.84
		Patient’s referral by an oncologist for receiving hospice services	2.58 ± 0.60
	Connection with other care settings	Establishing connection between hospice centers and other care settings (outpatient clinics and urban health centers, hospitals, and home)	2.73 ± 0.50
		Service coordination among different levels	2.70 ± 0.63
		Providing hospice centers with access to care and treatment documents	2.81 ± 0.78
		Creating a national hospice care network to record the data of patients receiving care	2.56 ± 0.84
		The connection of hospice care national network to the electronic health record system (SEPAS)	2.48 ± 0.78
**Specialist manpower**	Specialist interdisciplinary team	Providing interdisciplinary and team care	2.73 ± 0.50
		Training specialist hospice care delivery teams	2.58 ± 0.60
		The presence of a pain specialist in the hospice care delivery ream	2.73 ± 0.50
		The presence of a specialist nurse in the hospice care delivery team	2.58 ± 0.60
		The presence of a nurse as a coordinator of team care	2.91 ± 0.31
		The presence of a psychologist in the hospice care delivery team	2.73 ± 0.50
		The presence of a social worker in the hospice care delivery team	2.56 ± 0.84
		The presence of a spiritual caregiver/cleric in the hospice care delivery team	2.81 ± 0.78
		The presence of a general practitioner in the hospice care delivery team	2.91 ± 0.31
	skill and academic training/Expected competencies	Including the concept of hospice care in the curriculums of undergraduate nursing program and general practice	2.91 ± 0.31
		Including the concept of hospice care in the curriculums of the undergraduate programs in other related disciplines	2.56 ± 0.84
		Holding hospice care short-term skill training courses	3
		Holding ongoing training courses for the staff	2.73 ± 0.50
		Holding periodic hospice care workshops	2.46 ± 0.38
		Providing the necessary educational settings for training specialist manpower	2.64 ± 0.84
		Providing standard educational content for training specialist manpower	2.58 ± 0.60
		The involvement of related organizations in hospice care training	2.56 ± 0.84
		Defining credible specialized licenses and certificates for the trained manpower	2.58 ± 0.60
		Defining the competencies expected from the members of the hospice care team	2.81 ± 0.78
		Educational planning based on the competencies expected from the members of the hospice care team	2.58 ± 0.60
		Considering the topics related to hospice care in professional competence exams of nurses and physicians and other disciplines	2.56 ± 0.84
**Organizing the accountability system**	Transparent decision-making	Creating a joint taskforce for hospice care policymaking at the level of the Ministry of Health	2.73 ± 0.50
		Creating a joint taskforce for hospice care at the university level	2.91 ± 0.31
		Creating a joint taskforce for hospice care at the level of specialty hospitals	2.58 ± 0.60
		The participation of all sectors involved in the development of hospice care system	3
		The participation of governmental organizations in the development of hospice care system	2.73 ± 0.50
		The participation of private organizations (charities, NGOs, etc.) in the development of hospice care system	2.81 ± 0.78
		The formulation of job descriptions, roles, responsibilities and jurisdiction of each sector	3
	Accountable management	The presence of a system for monitoring, assessment and supervision	2.46 ± 0.38
		Developing indicators for monitoring, supervision and accreditation	2.46 ± 0.38
		Launching the electronic document system and the access to database to make decisions in the field of policymaking	2.56 ± 0.84
**Laying the groundwork *in the health system***	Optimal policymaking	Providing documents related to the importance of hospice care for health policymakers	2.91 ± 0.31
		Developing the national hospice care plan	2.85 ± 0.60
	Financial prerequisites	Government funding	2.68 ± 0.51
		Funding by private organizations	2.73 ± 0.50
		Developing insurance service packages	2.68 ± 0.51
		The involvement of charitable organizations in funding hospice services	2.73 ± 0.50
		Cost determination for secondary insurance services	2.34 ± 0.61
		Cost determination for governmental insurance services	2.68 ± 0.51
		Cost determination for services separately for each discipline	2.15 ± 0.43
		Supporting private sector in service provision	2.56 ± 0.84
		Insurance supportive policies for the proper insurance coverage of medications	2.73 ± 0.50
		Determining the cost-effectiveness of hospice services	2.91 ± 0.31
	Structural prerequisites	Providing infrastructure (resources, space, etc.)	2.83 ± 0.41
		Providing care equipment (ventilator, suction machine, oxygen, etc.)	2.85 ± 0.60
		Making structural changes in the health system	2.34 ± 0.67
		Access to cancer data registry	2.46 ± 0.38
	Legal prerequisites	Access to a variety of necessary narcotic drugs	2.68 ± 0.51
		Formulating guidelines for access to narcotics and necessary drugs	3
		The possibility for other disciplines such as nursing, pharmacy, etc. to prescribe drugs	2.34 ± 0.67
		Clarifying legal issues regarding hospice care	3
		Determining the legal steps regarding death at hospice centers	3
		Formulating guidelines and instructions in accordance with the law, ethics and scientific references in regard with continuing or stopping CPR	3
		Solving legal issues surrounding drug prescription (which drugs by whom?)	3
**Capacity building at the community**	Raising public awareness	Holding training courses at the community level	2.68 ± 0.51
		Providing the cultural and social infrastructure with the aim of increasing hospice care acceptability	3
	Research development	Holding annual hospice care seminars and conferences	2.85 ± 0.60

Accordingly, the practical process of establishing a hospice care delivery system for adult cancer patients were designed and approved by the research team (Table 5).

**TABLE 5:** The operational stages of establishing hospice care delivery system for adult patients with cancer.

At the level of the Ministry of Health and Medical Education
• Providing documents regarding the importance of establishing hospice care system and presenting it to the policymakers • Forming a joint taskforce for hospice care policymaking in the Ministry of Health • The development of national program and its general executive policies • The development of care service packages in hospice centers • The insurance coverage of hospice services, the necessary drugs and narcotics • Developing and approving guidelines and protocols for ethical decision-making < resuscitation at hospice centers and pain and symptom management • Defining the documents, the required specialized licenses and expected competencies for each discipline to provide services at hospice centers • Developing and approving supervisory rules • Communicating the national plan, executive policies, service packages, the formulated guidelines and protocols and the supervisory rules to medical universities and insurance organizations
**At the level of medical universities**
• Forming a joint taskforce for the establishment of hospice care system at the level of medical universities • Demographic needs assessment at the provincial level • Cost estimation and conduction of cost-effectiveness studies • Involving private sector, charitable organizations and NGOs • Formulation of hob descriptions and the jurisdiction of each sector • Allocating physical (space, equipment and medicine), financial and human resources • Forming hospice care delivery teams • The development and implementation of short-term skill training programs • Launching hospice centers (providing training and care delivery settings) • Developing and implementing programs at the community level to raise public awareness • Providing hospice centers and other service delivery settings with the access to SEPAS • Developing and communicating executive instructions (the referrer, the type of referral, transfer and admission at each level) to comprehensive health centers, long-term care centers, hospitals and educational and treatment centers affiliated to the university • Developing and communicating instructions on how to supply narcotics • Strategies for strengthening the family physician system and the referral system • Developing standard educational content and the integration of concepts into educational curriculums • Monitoring care provision at hospice centers
**At the level of comprehensive health centers, long-term care centers, hospitals and educational and medical centers**
• Forming a hospice care committee • Holding periodic and in-service courses of hospice care Offering 24/7 services • Implementing programs to raise public awareness • The implementation of communicated instructions regarding the establishment of hospice care delivery system • Conducting research and assessing the effectiveness of the provided services Providing feedback
**At the level of hospice centers**
• Providing 24/7 services and support to patients and families • Identifying the needs of the patient and family in all aspects through standard methods • Pain control and managing physical, mental, social and spiritual needs of the patients • Family management in mental, social and spiritual dimensions • The training and empowerment of the family Providing bereavement services • Formal communication with health service providers to identify patients in need of hospice care • Training service providers at hospice centers and other health service delivery settings • Public education at the community level Recording data, documentation and having access to SEPAS for sharing information • Conducting research and evaluating the provided services • Communication with policymaking taskforces at the university level • Developing plans for service development

## Discussion

In this study a model of hospice care delivery system for Iranian adult patients with cancer was developed and validated. According to the proposed model, only the establishment of hospice centers and care provision in these centers was prioritized. In regard with services provision, the management of patients’ physical and mental symptoms and offering the necessary information to the patient and the family are important and have high feasibility.

Consistent with the finding of this study, The Medicare Hospice Benefit program was established in 1982 and covers hospice care at home or inpatient facilities. According to the Dartmouth Atlas Project, 63% of patients with cancer enrolled in hospice before death in 2012 Dartmouth (Goodman et al., 2013). It should be noted that in Iran, even in the field of palliative care, services are more hospital-based, and home care centers are less developed due to the existing challenges (Mojen et al., 2017). On the other hand, given the current cultural background, most families seek care in inpatient centers (Valiee et al., 2012; Borhani et al., 2014; Heidari et al., 2018). In addition, recent Iranian studies on palliative care have focused on the need to launch various structures for providing care services, including hospice centers (Ansari et al., 2018; Khanali Mojen, 2018).

Based on our model, it is important in addition to manage physical, mental, spiritual, and social symptoms of the patient, provide mental, spiritual and social support for the family, and offer bereavement care. Like the model designed in this study, based a designed Season’s Community-Based Palliative care model In Western North Carolina, an inter-disciplinary team provided care to patients in both outpatient and inpatient settings, including psychosocial/spiritual care, advanced care planning, symptom management, and patient/family education. According to developed model, spiritual support is also one of the domains of comprehensive care in hospice centers and has a high feasibility. Kang (2018) stated based on the study of different models, spiritual care is one of the core components of hospice palliative care. The literature has supplied numerous evidence-based models (such as quality of life, coping, the spiritual-relational view, …) that focus on spiritual care and can be used as models for providing spiritual care in these centers. The above results confirm the results of the present study (Kang, 2018).

Obviously, the need for bereavement care is a priority which has high feasibility in this model, too. Consistent with this finding, bereavement care is an essential component of hospice care that includes anticipating grief reactions and providing ongoing support for the bereaved over a period of 13 months. While the terms are often used interchangeably, bereavement refers to the state of loss, and grief is the reaction to loss (Grant et al., 2020). The results of the previous Iranian studies on palliative care also confirm this finding (Khanali Mojen, 2018; Pakseresht et al., 2018). Based on the designed model in the domain *comprehensive care plan*, participatory decision-making and consensus in the definition, have priority. These patients need to receive comprehensive and standard care for their quality of life to improve during their remaining days. Providing this type of care requires the development of guidelines and instructions that are part of the palliative care delivery process. Highlighting the structures and the process of care is very important. In addition, the development of care guidelines and standards, and service packages tailored to the needs of the family is of great significance (Eshaghian-dorcheh et al., 2020).

In the developed model, the integration of services at the primary, secondary and the tertiary levels of the health system was feasible. In addition, according to the designed model, hospice centers will be connected with other care settings. In line with the above-mentioned, it should be noted that the studies have shown that the palliative care and hospice services are provided mainly at the primary health care (PHC) (Zarea et al., 2020). As suggested by WHO, providing palliative care at the PHC level is mandatory, in line with UHC goals (World Health Organization, 2018a). Therefore, the integration of services into this level of care is essential in developing a hospice care delivery system. Consistent with this study, Hojjat-Assari’s model, palliative care is provided at various levels of the health system (Hojjat-Assari et al., 2021).

Furthermore, in this model patients will be referred by oncologists, general practitioners, family physicians, and nurses. As you see, the patient referral process in this model is reversed so that sources of referral are central and specialist units and general hospitals. These findings are in line with model, and Khanali’s model in which patients are referred from specialist centers to the palliative care clinics (Mojen et al., 2017). It seems that the specialty-oriented culture dominant in the Iranian society and the patient’s visiting specialists even for the most minor issues, the public and caregivers’ insufficient knowledge and awareness, palliative service delivery’s being a novel care approach, the shortage or lack of different settings which offer this type of service, easier access to specialized services and more facilities in the secondary and tertiary levels of the health system in Iran, as well as the above mentioned items are the main reasons for which the experts have allocated higher scores to the indicator *patients’ referral by oncologists to receive hospice services*. Referral by an oncologist may be effective in providing the infrastructures for the establishment of a hospice care system and a starting point for the provision of this type of service at the community.

In the developed model, it was also determined that the hospice care team should at least include a general practitioner, a trained nurse, and a spiritual caregiver/cleric. Psychologists and social workers came next. These results are in line with WHO guidelines (World Health Organization, 2016). Furthermore, the spiritual caregiver is a key member of the hospice care team according to the cultural-religious background of the country.

Contrary to the results of other studies in the field of palliative care in Iran, using volunteer forces for the provision of hospice services was not a priority. It should be noted that volunteers and charity donors are active members of the palliative care system in many countries around the world, and the provision of many services, especially hospice care, depends on their participation (Hunter and Orlovic, 2018). In Iran, most volunteers work in charities and in the field of community services, especially psychological and financial support (Ansari et al., 2018).

In regard with academic education and the expected skills and competencies, according to the experts, the development of the specialized discipline, the fellowship course, and master’s degree program for hospice care are not mandatory. *Holding short-term hospice care skill training courses* to provide specialist manpower obtained higher feasibility, which is in line with the policies adopted in other countries around the world and even in other Middle Eastern countries (Barasteh et al., 2020).

On ***organizing the accountability system***, In the developed model, *the formation of a hospice care policymaking taskforce at the university level*, *the formation of a hospice care committee at the hospital level*, *the participation of all the sectors involved in hospice care delivery, including private and public sectors*, and *the formulation of job descriptions, roles, responsibilities and the jurisdiction of each department* were of great feasibility. In line with our model, Ansari’s palliative care model in Iran also shows that the formation of executive committees at various levels of the Ministry of Health, including the Deputy Minister of Education, universities, and hospitals will play an important role in the development of palliative care services (Ansari et al., 2019).

Laying the groundwork *in the health system* in our developed model is possible in the following ways. *Providing documents in regard with the importance of hospice care for health policymakers* and *developing a national hospice care plan*, which is regarded as the official starting point for the provision of these services in many communities (Zarea et al., 2020). Another important point in the designed model is providing financial prerequisites through various funding approaches including public funding, private funding, insurance coverage, development of service packages and setting service costs, as well as supporting charitable organizations. However, funding through the private sector and seeking support from donors seems to be more important. Privatization in the health system can lead to positive outcomes. More funding and investments in the private sector, flexibility, and the freedom of managers in utilizing and making better use of the available funds will enable them to perform more activities with more diversity to increase patients and families’ satisfaction, as well as providing higher quality services (Eshaghian-dorcheh et al., 2020). Since governments alone cannot address the growing needs of cancer patients, charities and volunteers can be of great help to the government and patients at the community level, provided that all the activities are monitored and organized based on the set rules and regulations (Groeneveld et al., 2017).

In the present model, making plans to meet the structural prerequisites of care delivery in hospice centers, including physical space and supportive care equipment, is considered a priority and has high feasibility. This factor is one of the principles of designing every health care system, which is line with the results of other studies (Rassouli and Sajjadi, 2016; Barasteh et al., 2020).

Based on this model, the greatest need in regard with the legal and ethical requirements of care in hospice centers is the development of guidelines for administering narcotics and necessary medications and clarifying legal issues related to care, drug prescription, death, and resuscitation operations in these centers. Developed systems of palliative, hospice and end-of-life care around the world have clear rules regarding euthanasia, DNR, and decision making based on patients’ interests (Economist Intelligence Unit, 2015).

In regard with research and public awareness, there is an evident need for providing cultural and social infrastructures to increase the acceptance of hospice care and conduct cost-effectiveness studies, which has the highest level of feasibility based on our model. Given the growing rate of research in the field of palliative care in Iran in recent years, and cancer-related research centers and journals’ prioritizing the publication of articles in the field of palliative care, it is important to create proper infrastructures in the existing centers, determine research priorities in this area, and finance further research (Rassouli and Sajjadi, 2014).

Due to the limited number of palliative care experts in Iran, there were few knowledgeable individuals participating in the surveys. On the other hand, cultural, social, and religious factors are reported as underlying factors in the development of the care system. However, given that Iran has a wide cultural diversity, this issue may limit the generalization of the research findings. Therefore, its pilot implementation in different regions is recommended.

## Conclusion

The aim of this study was to develop and validate a model for establishing a hospice care delivery system for the adult patients with cancer in Iran. Based on the developed model, it was discovered that the Iranian health system needs to launch hospice centers and provide comprehensive services to cancer patients and their families. It is also necessary to design a comprehensive care plan and train specialist manpower in order to address this need. In addition, the integration of these services into the health system and organizing the accountability system before the establishment of this type of services is an inevitable necessity. To this end, policy making through providing the infrastructure in the health system, and capacity-building at the community is also of great importance. Therefore, in order to implement the developed model in Iran, it is necessary to consider the following items: creating job titles in regard with these services especially for physicians and nurses, implementing the program with the presence of community health nurses and family physicians based on the referral process according to the similar and approved instructions, formulating clinical guidelines and the necessity of its implementation at all levels, encouraging the private sector and NGOs for investment, developing service packages for palliative hospice care and end-of-life care based on the insurance costs, and developing the indicators of quality care to audit in this field.

By proposing a model tailored to the structure of the Iranian health system, in addition to being applicable in policymaking, launching relevant services in the country, and developing palliative care network. It can be used in various educational, research and clinical fields including hospice care specialty courses, the design and implementation of pilot studies and the management of end-stage cancer patients and their families in different health system settings.

## Author’s Note

This manuscript was a part of a Ph.D. dissertation in nursing at the Chronic Disease Care Research Center affiliated to the Ahvaz Jundishapur University of Medical Sciences.

## Data Availability Statement

The original contributions presented in the study are included in the article/supplementary material, further inquiries can be directed to the corresponding author/s.

## Ethics Statement

The studies involving human participants were reviewed and approved by the Ahvaz Jundishapur University of Medical Sciences under the code of IR.AJUMS.REC.1397.306. Participants provided their written informed consent to participate in the study.

## Author Contributions

SB and KZ conceived the study and contributed to its design. SB did the quantitative and qualitative data collection, analysis, and interpretation and drafted the manuscript. MR, MH, and SM contributed to study design, draft preparation, and study coordination. KZ, MR, MH, and SM supervised and coordinated the study project. SH participated in study consultation and critical revision of the article. All authors read and approved the final manuscript.

## Conflict of Interest

The authors declare that the research was conducted in the absence of any commercial or financial relationships that could be construed as a potential conflict of interest.

## Publisher’s Note

All claims expressed in this article are solely those of the authors and do not necessarily represent those of their affiliated organizations, or those of the publisher, the editors and the reviewers. Any product that may be evaluated in this article, or claim that may be made by its manufacturer, is not guaranteed or endorsed by the publisher.

## Footnotes

1 Intensive Care Units (ICUs).

2 Emergency Rooms (ERs).

## References

[B1] Ansari M. (2018). Developing Palliative Care System for Adult with Cancer, Faculty of Nursing and Midwifery, Shahid Behashti University of Medical Sciences, Tehran, Iran.

[B2] Amiresmaeili M. R. Imani E. Sarvestani J. (2015). Evaluation of terminal life cost for patients admitted in teaching hospitals affiliated with kerman university of medical sciences in 2014. *J. Health Based Res.* 1 133–143.

[B3] Angus D. C. Truog R. D. (2016). Toward better ICU use at the end of life. *JAMA* 315 255–256. 10.1001/jama.2015.18681 26784767

[B4] Ansari M. Rassouli M. Akbari M. E. Abbaszadeh A. Akbarisari A. Haghighat S. (2019). Process challenges in palliative care for cancer patients: a qualitative study. *Middle East J. Cancer* 10 43–53.

[B5] Ansari M. Rassouli M. Akbari M. E. Abbaszadeh A. Akbarisari A. (2018). Palliative care policy analysis in Iran: a conceptual model. *Indian J. Palliat. Care* 24:51. 10.4103/IJPC.IJPC_142_17 29440807 PMC5801630

[B6] Azami-Aghdash S. Ghojazadeh M. Aghaei M. H. Naghavi-Behzad M. Asgarlo Z. (2015). Perspective of patients, patients’ families, and healthcare providers towards designing and delivering hospice care services in a middle income country. *Indian J. Palliat. Care* 21:341. 10.4103/0973-1075.164898 26600704 PMC4617043

[B7] Barasteh S. Rassouli M. Parandeh A. Vahedian-Azimi A. Zaboli R. Khaghanizadeh M. (2020). Palliative care in the health system of Iran: a review of the present status and the future challenges. *Asian Pac. J. Cancer Prev.* 21:845. 10.31557/APJCP.2020.21.3.845 32212816 PMC7437322

[B8] Borhani F. Hosseini S. Abbaszadeh A. (2014). Commitment to care: a qualitative study of intensive care nurses’ perspectives of end-of-life care in an I slamic context. *Int. Nurs. Rev.* 61 140–147. 10.1111/inr.12079 24382147

[B9] Bray F. Ferlay J. Soerjomataram I. Siegel R. L. Torre L. A. Jemal A. (2018). Global cancer statistics 2018: GLOBOCAN estimates of incidence and mortality worldwide for 36 cancers in 185 countries. *CA Cancer J. Clin.* 68 394–424. 10.3322/caac.21492 30207593

[B10] Clark D. Armstrong M. Allan A. Graham F. Carnon A. Isles C. (2014). Imminence of death among hospital inpatients: prevalent cohort study. *Palliat. Med.* 28 474–479. 10.1177/0269216314526443 24637342 PMC4845030

[B11] Eshaghian-dorcheh A. Zandi M. Rasouli M. Tahmasebi M. Esmaielzadeh F. (2020). Evaluating the cost-effectiveness of home-based palliative care for children with special health care needs: a review study. *Int. J. Pediatr.* 8 12381–12395.

[B12] Goodman D. C. Morden N. E. Chang C. H. Fisher E. S. Wennberg J. E. (2013). *Trends in Cancer Care Near the End of Life. A Dartmouth Atlas of Health Care Brief.* Lebanon, NH: The Dartmouth Institute for Health Policy and Clinical Practice. 36441862

[B13] Grant M. Hudson P. Forrest A. Collins A. Israel F. (2020). Developing a model of bereavement care in an adult tertiary hospital. *Aust. Health Rev.* 45 110–116. 10.1071/AH19270 33353586

[B14] Groeneveld E. I. Cassel J. B. Bausewein C. Csikós Á Krajnik M. Ryan K. (2017). Funding models in palliative care: lessons from international experience. *Palliat. Med.* 31 296–305. 10.1177/0269216316689015 28156188 PMC5405831

[B15] Habibi A. Sarafrazi A. Izadyar S. (2014). Delphi technique theoretical framework in qualitative research. *Int. J. Eng. Sci* 3 8–13.

[B16] Heidari M. R. Hosseinkhani S. N. Norouzadeh R. (2018). Elderly’s attitude toward end-of-life concerns. *J. Nurs. Educ.* 7 35–44.

[B17] Hojjat-Assari S. Rassouli M. Madani M. Heydari H. (2021). Developing an integrated model of community-based palliative care into the primary health care (PHC) for terminally ill cancer patients in Iran. *BMC Palliat. Care* 20:100. 10.1186/s12904-021-00795-2 34182980 PMC8240381

[B18] Seniors Health Research Transfer Network (2012). *Hospice Palliative Care System Design Framework for Developing Regional Systems of Hospice Palliative Care: a Provincial Framework [Online].* Available online at: http://www.hpco.ca/wp-content/uploads/HPC-System-Design-Framework.pdf (accessed February 20, 2018).

[B19] Hui D. (2014). Definition of supportive care: does the semantic matter? *Curr. Opin. Oncol.* 26 372–379. 10.1097/CCO.0000000000000086 24825016 PMC12034444

[B20] Hunter J. Orlovic M. (2018). *End of Life Care in England: A Breefing Paper.* London: Institude for Public Policy Research.

[B21] Khanali Mojen L. (2018). *Designing Palliative Care System for Pediatrics with Cancer in Iran, Faculty of Nursing and Midwifery, Shahidbehashti University of Medical Sciences, Tehran, Iran.*

[B22] Kang K.-A. (2018). Models for spiritual care in hospice and palliative care. *Korean J. Hosp. Palliat. Care* 21 41–50. 10.14475/kjhpc.2018.21.2.41

[B23] Kumar P. Wright A. A. Hatfield L. A. Temel J. S. Keating N. L. (2017). Family perspectives on hospice care experiences of patients with cancer. *J. Clin. Oncol.* 35:432. 10.1200/JCO.2016.68.9257 27992271 PMC5455697

[B24] Mojen L. K. Rassouli M. Eshghi P. Sari A. A. Karimooi M. H. (2017). Palliative care for children with cancer in the Middle East: A comparative study. *Indian J. Palliat. Care* 23:379. 10.4103/IJPC.IJPC_69_17 29123342 PMC5661338

[B25] National Hospice and Palliative Care Organization (2020). *NHPCO Releases New Facts and Figures Report on Hospice Care in America.* Alexandria, VA: National Hospice and Palliative Care Organization.

[B26] Nemati S. Rassouli M. Ilkhani M. Baghestani A. R. (2018). Perceptions of family caregivers of cancer patients about the challenges of caregiving: a qualitative study. *Scand. J. Caring Sci.* 32 309–316. 10.1111/scs.12463 28869659

[B27] Pakseresht M. Baraz S. Rasouli M. Reje N. Rostami S. (2018). A comparative study of the situation of bereavement care for children with cancer in Iran with selected countries. *Int. J. Pediatr.* 6 7253–7263.

[B28] Rassouli M. Sajjadi M. (2014). *Palliative Care in the Islamic Republic of Iran. Palliative Care to the Cancer Patient: The Middle East as a Model for Emerging Countries.* New York: Nova Scientific Publisher, 39.

[B29] Rassouli M. Sajjadi M. (2016). Palliative care in Iran: moving toward the development of palliative care for cancer. *Am. J. Hosp. Palliat. Med.* 33 240–244. 10.1177/1049909114561856 25492970

[B30] Rauch W. (1979). The decision delphi. *Technol. Forecast. Soc. Change* 15 159–169. 10.1016/b978-0-444-81485-2.50017-4

[B31] Valiee S. Negarandeh R. Dehghan Nayeri N. (2012). Exploration of Iranian intensive care nurses’ experience of end-of-life care: a qualitative study. *Nurs Crit. Care* 17 309–315. 10.1111/j.1478-5153.2012.00523.x 23061621

[B32] World Health Organization (2016). *Planning and Implementing Palliative Care Services: A Guide For Programme Managers.* Geneva: World Health Organization.

[B33] World Health Organization (2018a). *Integrating Palliative care and Symptom Relief Into Paediatrics: A WHO Guide for Health-Care Planners, Implementers and Managers.* Geneva: World Health Organization.

[B34] World Health Organization (2018b). *Noncommunicable Diseases Country Profiles 2018.* Geneva: World Health Organization.

[B35] Wright A. A. Keating N. L. Ayanian J. Z. Chrischilles E. A. Kahn K. L. Ritchie C. S. (2016). Family perspectives on aggressive cancer care near the end of life. *JAMA* 315 284–292. 26784776 10.1001/jama.2015.18604 PMC4919118

[B36] Zarea K. Rassouli M. Hazrati M. Molavynejad S. Beiranvand S. (2020). Comparison of The hospice palliative care delivery systems in iran and selected countries. *Int. J. Cancer Manage* 13 1–17. 10.1186/s13054-016-1208-6 27885969 PMC5493079

[B37] Ziloochi M. H. Pourreza A. Akbari F. Rahimi F. A. (2012). Evaluating the hospitalization costs for elderly patients in teaching hospitals of Kashan university of medical sciences during 2009-10. *Feyz* 16 86–94.

